# Targeting XPO1 and PAK4 in 8505C Anaplastic Thyroid Cancer Cells: Putative Implications for Overcoming Lenvatinib Therapy Resistance

**DOI:** 10.3390/ijms21010237

**Published:** 2019-12-29

**Authors:** Husain Yar Khan, James Ge, Misako Nagasaka, Amro Aboukameel, Gabriel Mpilla, Irfana Muqbil, Mark Szlaczky, Mahmoud Chaker, Erkan Baloglu, Yosef Landesman, Ramzi M. Mohammad, Asfar S. Azmi, Ammar Sukari

**Affiliations:** 1 Department of Oncology, Wayne State University School of Medicine, Detroit, MI 48201, USA; khanh@karmanos.org (H.Y.K.); jamesge13@outlook.com (J.G.); nagasakm@karmanos.org (M.N.); kameelo@karmanos.org (A.A.); mpillag@karmanos.org (G.M.); szlaczky@karmanos.org (M.S.); mahc1992@gmail.com (M.C.); mohammad@karmanos.org (R.M.M.); azmia@karmanos.org (A.S.A.); 2 Department of Chemistry and Biochemistry, University of Detroit Mercy, Detroit, MI 48221, USA; muqbilir@udmercy.edu; 3 Restorbio Inc., Boston, MA 02116, USA; ebaloglu@restorbio.com; 4 Karyopharm Therapeutics Inc., Newton, MA 02459, USA; yosef@karyopharm.com

**Keywords:** thyroid cancer, lenvatinib, therapy resistance, selinexor, PAK4 inhibitor, selective inhibitors of nuclear export

## Abstract

Lenvatinib is a multitargeted tyrosine kinase inhibitor (TKI) that shows improved median progression-free survival (PFS) in patients with thyroid carcinomas. However, virtually all patients ultimately progress, indicating the need for a better understanding of the mechanisms of resistance. Here, we examined the molecular profile of anaplastic thyroid cancer cells (8505C) exposed to lenvatinib and found that long-term exposure to lenvatinib caused phenotypic changes. Consistent with change toward mesenchymal morphology, activation of pro-survival signaling, nuclear exporter protein exportin 1 (XPO1) and Rho GTPase effector p21 activated kinases (PAK) was also observed. RNA-seq analysis showed that prolonged lenvatinib treatment caused alterations in numerous cellular pathways and several oncogenes such as *CEACAM* (carcinoembryonic antigen-related cell adhesion molecule) and *NUPR1* (Nuclear protein 1) were also upregulated. Further, we evaluated the impact of XPO1 and PAK4 inhibition in the presence or absence of lenvatinib. Targeted inhibition of XPO1 and PAK4 could sensitize the 8505C cells to lenvatinib. Both XPO1 and PAK4 inhibitors, when combined with lenvatinib, showed superior anti-tumor activity in 8505C sub-cutaneous xenograft. These studies bring forward novel drug combinations to complement lenvatinib for treating anaplastic thyroid cancer. Such combinations may possibly reduce the chances of lenvatinib resistance in thyroid cancer patients.

## 1. Introduction

Thyroid cancer remains a significant health problem affecting half a million individuals worldwide. According to the American Cancer Society’s most recent estimates, in the United States, about 52,070 new cases of thyroid cancer (14,260 in men and 37,810 in women) will be diagnosed and about 2170 deaths from thyroid cancer (1020 men and 1150 women) will occur in 2019 (American Cancer Society. Cancer Facts & Figures 2019; https://www.cancer.org/cancer/thyroid-cancer/about/key-statistics.html). More than 90% of thyroid cancer (TC) are differentiated thyroid carcinomas (DTCs) that arise from follicular cells [papillary thyroid cancer (PTC)-90%, follicular thyroid cancer (FTC)-10%], while medullary thyroid cancer (MTC) and anaplastic thyroid cancer (ATC) account for less than 5% and 2%, respectively, of all thyroid cancers [1]. For the majority of patients, complete thyroidectomy is the treatment of choice [2]. Additionally, radioiodine is suggested in high-risk patients and considered in intermediate risk DTC patients [2]. Nevertheless, DTC tumor cells often times lose the iodide uptake ability, thereby becoming resistant to radioiodine therapy [3]. In these patients there is a significant worsening of the prognosis. Due in part to the lack of effective agents for aggressive and metastatic DTC and MTC, there is a huge drive to identify novel drugs for these disease subtypes. Next generation sequencing and large-scale patient profiling have revealed several genetic alterations in different molecular pathways that drive TC disease development, progression, and therapy resistance [4]. Mutations and rearrangements in proto-oncogene (RET)/PTC, BRAF, RAS, and vascular endothelial growth factor receptor 2 (VEGFR-2), as well as alterations in angiogenesis pathway, are prominently observed during the development of TC [5]. A significant proportion of TC sub-types show aberrations in the receptor tyrosine kinase pathways giving traction to the use of Tyrosine kinase inhibitor (TKI) based therapies for aggressive TC, including DTC, MTC, and ATC [6]. TKIs demonstrated evaluable clinical responses and stabilization of disease and drugs such as vandetanib and cabozantinib have been approved for the treatment of MTC, while sorafenib and lenvatinib have been approved for DTC that is refractory to radioiodine [7].

Lenvatinib (E7080) is an oral, multitargeted TKI of VEGFR-1, -2, and -3, FGFR-1, -2, -3, and -4, PDGFR α, RET, and KIT [8]. Lenvatinib is approved for the treatment of radioiodine-refractory differentiated thyroid cancer [9]. In a Phase III study, patients showed improved median PFS (18.3 months) in the lenvatinib group than in the placebo one (3.6 months; *p* < 0.001). In the lenvatinib group, there were 4 CR and 165 PR, with a response rate of 64.8% versus 1.5% in the placebo group (*p* < 0.001). While lenvatinib prolongs median progression-free survival, median overall survival was not reached in either group and side effects were common [10]. Also, virtually all patients will eventually progress on TKIs. These observations indicate that: (a) there is a lack of understanding in our knowledge of the impact of RTKI in thyroid carcinoma as: (b) not much is known on the underlying resistance mechanisms to lenvatinib or related RTKIs. In this report we evaluated the resistance mechanism by creating a lenvatinib resistant anaplastic thyroid cancer cell line which was grown in long term lenvatinib culture conditions. Furthermore, we showed that targeted inhibition of XPO1 and PAK4 could sensitize anaplastic thyroid cancer cells to lenvatinib.

## 2. Results

### 2.1. Development of Lenvatinib Resistant Cell Line

**Figure 1:**
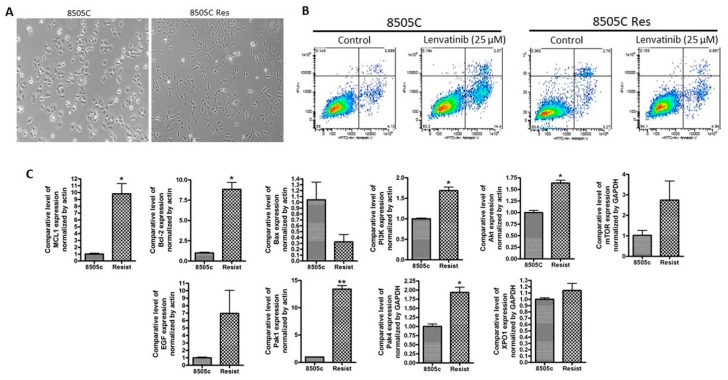
Development of lenvatinib resistant thyroid cancer cell line. 8505C human thyroid carcinoma (undifferentiated) cell line was grown in culture media containing 25 μM lenvatinib for 72 days. Cells were passaged twice a week with drugs added to media continuously. (**A**) Photomicrographs (10× magnification) showing emergence of mesenchymal morphology in the lenvatinib exposed cells. (**B**) The resulting lenvatinib resistant cell line 8505C Res and parent 8505C were seeded in 6 well plates at a density of 50,000 cells per well. After 24 h cells were exposed to control (DMSO) or lenvatinib (25 μM) for 72 h. Annexin V FITC apoptosis analysis was performed according to the manufacturer’s protocol (Biovision). (**C**) RT-PCR analysis for the changes in expression of markers related to apoptosis signaling, PI3K signaling and EGF. Expression values were normalized to actin or GAPDH. * *p* < 0.05; ** *p* < 0.01

In order to mimic the lenvatinib resistance, we cultured 8505C cell line in media containing 25 µM lenvatinib for 72 days. An analysis of morphology of the 8505C lenvatinib resistant (8505C Res) cell line demonstrated a change from epithelial to mesenchymal phenotype (Figure 1A). More significantly, at the end of the treatment period we tested the cells for apoptosis induction. Compared to parent 8505C cells, which showed apoptosis upon treatment with 25 µM lenvatinib, apoptosis induction was less in the 8505C Res cells at the same dose of lenvatinib (Figure 1B). We further characterized the mRNA expression of different markers in parent vs resistant cell lines using RT-PCR. As can be seen from the results of Figure 1C, compared to parent cell line, the resistant cells showed a marked increase in the expression of pro-survival markers including Mcl-1 and Bcl-2, and reduction in pro-apoptotic marker Bax. Additionally, we also observed enhancement in the expression of PI3K, AKT and mTOR alongside the activation of downstream molecules such as Rho GTPase effector p21 activated kinases (PAKs), particularly PAK1 and PAK4. Interestingly, nuclear exporter protein XPO1, also known as the chromosome region maintenance 1 (CRM1), was found to be activated in the lenvatinib resistant cells.

### 2.2. Molecular Analysis of EMT and Stemness Markers in Lenvatinib Resistant Cells

**Figure 2:**
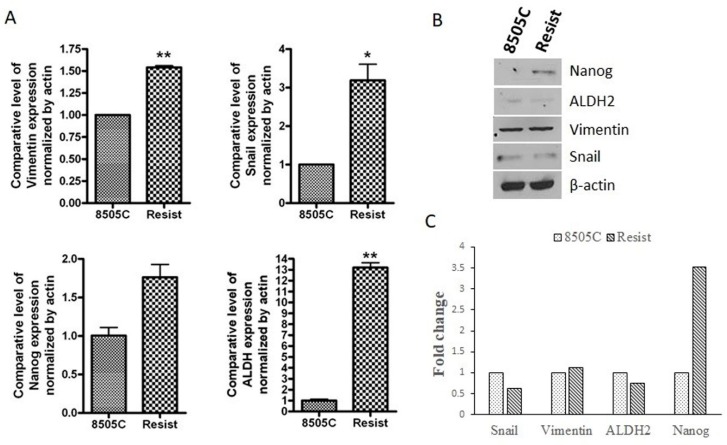
Molecular analysis of EMT and stemness markers in resistant cells. 8505C thyroid cancer cells were treated with 25 µM lenvatinib for 72 days (labeled as Resist). (**A**) The total RNA from these cells and the parent 8505C cells was extracted and subjected to RT-PCR analysis. Expression values were normalized to actin. * *p* < 0.05; ** *p* < 0.01 (**B**) Total protein was extracted from the parent and resistant 8505C cells and protein concentration was determined as described in the Methods. 30 µg of protein lysates were subjected to western blotting using antibodies against Snail, Vimentin, ALDH2 and Nanog. β-actin was used as a loading control. (**C**) Expression of each protein was indicated as fold change relative to the control and quantitative analysis of mean pixel density of the blots was performed using ImageJ software.

Given that epithelial-to-mesenchymal transition is an inherent property of stem-like cells, we next evaluated the expression of EMT and stem cell markers in the mesenchymal resistant cells. As can be seen from the results of Figure 2A, the resistant cells showed marked increase in RNA levels of mesenchymal markers *Snail* (*p* < 0.05) and *Vimentin* (*p* < 0.01). RNA levels of classical stem cell markers *ALDH* (*p* < 0.01) and *Nanog* (ns) were also observed to be elevated in resistant cells. However, when protein expression of these mesenchymal and stemness markers was examined, only the expression of Nanog was found to be considerably elevated (3.5-fold increase) compared to the parent 8505C cells (Figure 2B,C). There was only a slight increment in Vimentin expression, while Snail and ALDH2 expression turned out to be less than that in the parent cell line (Figure 2C). Since the results from the RNA and protein expression analyses of the abovementioned markers do not align completely with each other, the emergence of EMT and stemness in the resistant cells cannot be established conclusively.

### 2.3. Transcriptomic Analysis of the Parent and Resistant Cell Pair

**Figure 3:**
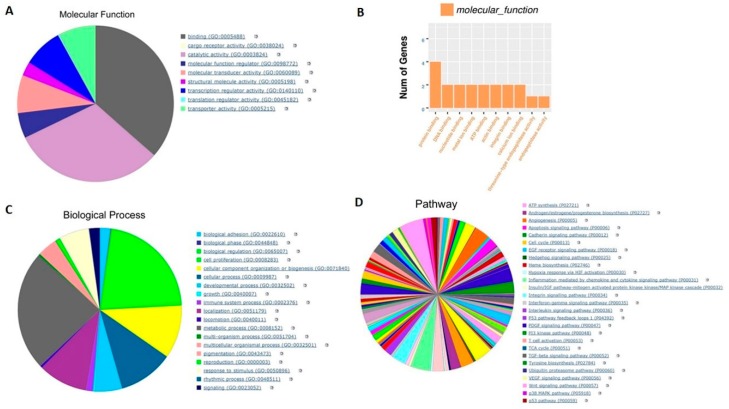
Transcriptomic analysis of the 8505C and 8505C Res cells. 8505C thyroid cancer cells were continuously cultured with 25 μM lenvatinib for 72 days. The total RNA from these cells was extracted and subjected to RNA-seq analysis. (**A**) Molecular function analysis of the statistically significant transcript changes in parent vs resistant cells. (**B**) Number of genes altered. (**C**) Biological process associated with statistically significant gene changes. (**D**) Pathways associated with the biological processes altered in the resistant cell compared to the parent. Pie charts and the table in this figure represent combined analysis for the 8505C cell pair.

We performed RNA-seq analysis to evaluate the changes in the transcription of parent 8505C cells and their resistant counterparts. As can be seen from the results of Figure 3A,B, the gene annotation analysis of molecular function showed that prolong treatment of cells with lenvatinib mainly regulated binding function including protein, DNA, nucleotide, ATP and other binding and catalytic activity. Likewise, the gene annotation analysis of biological processes demonstrated that lenvatinib treatment majorly affected metabolic process and biological regulation (Figure 3C). Further, the gene annotation analysis of pathway revealed that a total of 155 pathways including those associated with angiogenesis, apoptosis, cell cycle, and inflammation were modulated (Figure 3D). Moreover, consistent with the morphology change (shown in Figure 1A), several EMT markers such as *Snail1*, *Snail2* and *ZEB1* were found to be up-regulated in 8505C Res cells (Table 1). Also, as can be noted from Table 1, several oncogenes such as *CEACAM* and *NUPR1* were up-regulated in 8505C Res thyroid cancer cells, suggesting cell resistance to lenvatinib treatment.

### 2.4. Targeted Inhibition of XPO1 or PAK4 in Combination with Lenvatinib Causes Synergistic Inhibition of Thyroid Cancer Cell Growth

**Figure 4:**
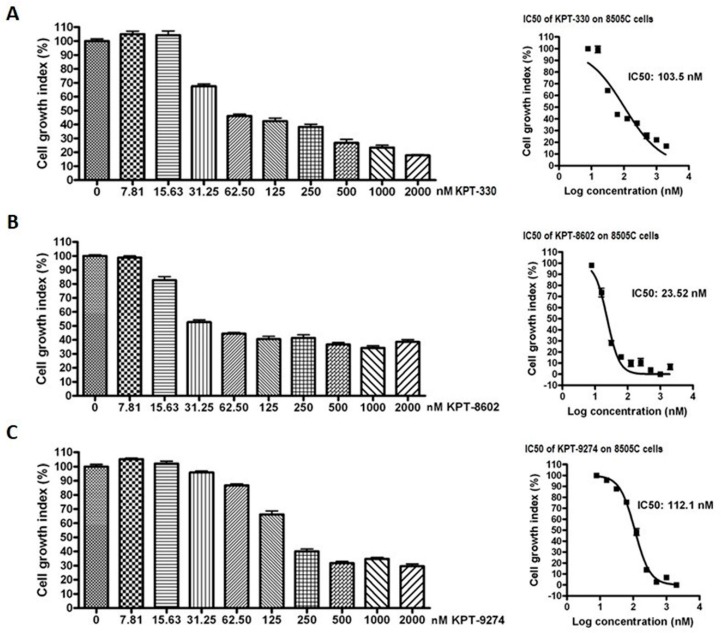
Inhibitors of XPO1 and PAK4 suppress thyroid cancer cell proliferation in vitro. 8505C cells were seeded in 96 well plates at a density of 5000 cells per well. The next day cells were exposed to increasing concentrations of either (**A**) KPT-330, (**B**) KPT-8602 or (**C**) KPT-9274 for 72 h. At the end of treatment, MTT assay was performed according to the standard procedure described in methods section. Each point represents six replicates.

We next explored the impact of targeting nuclear exporter protein XPO1 and Rho GTPase effector PAK4 either alone or in combination with lenvatinib in anaplastic thyroid cancer cells. As can be seen in Figure 4, XPO1 inhibitors KPT-330 and KPT-8602, as well as PAK4-NAMPT dual inhibitor KPT-9274, could suppress the proliferation of 8505C cells in a concentration-dependent manner. The IC50s of these compounds were in the low nanomolar range (ranging from 23.52 nM to 112.1 nM). Further, we evaluated the effect of inhibiting PAK1 on anaplastic thyroid cancer cells by treating 8505C cells with a PAK1 inhibitor IPA-3. Interestingly, PAK1 inhibition did not show any cell growth inhibition at the tested concentrations (Figure S1A).

**Figure 5:**
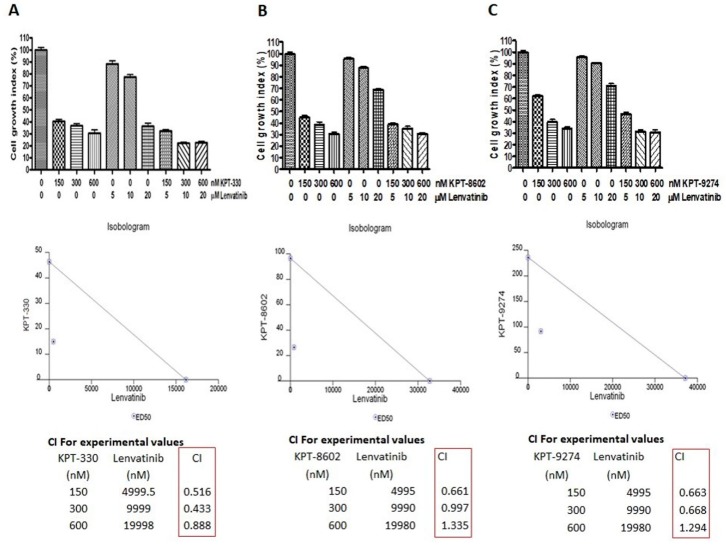
Targeting nuclear transport machinery and Rho GTPase effectors to sensitize thyroid cancer cells to lenvatinib. 8505C thyroid cancer cells were treated with indicated concentrations of either lenvatinib or (**A**) KPT-330, (**B**) KPT-8602 or (**C**) KPT-9274 or combinations of lenvatinib with KPT-330/KPT-8602/KPT-9274 for 3 days. Cell proliferation assay using MTT was conducted. Based on the cell proliferation data, isobologram analysis was performed to calculate the combination index (CI) using CalcuSyn software.

Next, we evaluated the impact of combined treatment of each of these inhibitors with lenvatinib on the proliferation of 8505C cells. As shown in Figure 5, the combination treatments resulted in synergistic inhibition of 8505C cell proliferation. The isobologram analysis further revealed a combination index value of less than 1 (CI < 1), which is indicative of a synergistic effect, for KPT-330 + lenvatinib at all dose combinations tested. In addition, for KPT-8602 + lenvatinib and KPT-9274 + lenvatinib combinations, synergy (CI < 1) was also observed at all dose combinations except one (Figure 5). These results indicate that the targeted inhibition of Rho GTPase effector or nuclear exporter proteins can sensitize thyroid cancer cells to lenvatinib treatment. In addition, when the effect of PAK1 inhibitor IPA-3 in combination with lenvatinib on the growth of 8505C cells was assessed, no synergistic inhibition was observed (Figure S1B). Since, IPA-3 selectively inhibits PAK1 and does not cause inhibition of groups II PAKs (including PAK4), these results suggest that while targeting PAK4 in anaplastic thyroid cancer cells is a viable option, targeting PAK1 is not.

### 2.5. XPO1 or PAK4 Inhibition Sensitizes Thyroid Cancer Sub-Cutaneous Xenograft to Lenvatinib

**Figure 6:**
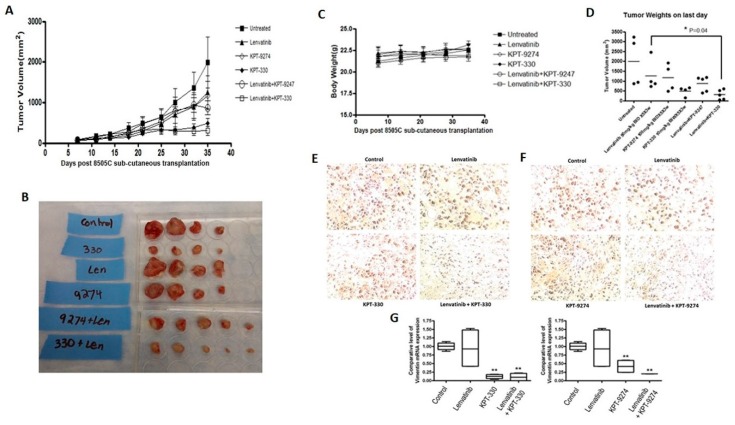
In vivo anti-tumor activity of novel combinations. (**A**) The mice carrying 8505C thyroid cancer cell xenografts were treated with either lenvatinib, KPT-330, KPT-9274 or a combination of lenvatinib with KPT-330/KPT-9274 and the effect of these treatments on tumor volume was assessed up to 35 days post-transplantation. (**B**) Photograph showing changes in the size of tumors harvested from different mice groups at the end of the experiment. (**C**) Body weight changes during the course of the treatment. (**D**) Gross tumor weight at the end of the treatment period. (**E**,**F**) Immunostaining for ki67 of the tissue sections of tumors harvested from untreated mice as well as from those receiving different treatments. Images were taken at 200× magnification. (**G**) RT-PCR analysis on RNA isolated from tumors showing comparative expression of *Vimentin* mRNA in various treatment groups. ** *p* < 0.01

Encouraged by our results from the synergy analysis, we next evaluated the impact of combined treatment of lenvatinib with either KPT-9274 or clinical grade KPT-330 (XPOVIO tablets containing orally available KPT-330 as the active ingredient) in sub-cutaneous xenograft developed from 8505C cells. As shown in Figure 6A,B, lenvatinib administered at 50 mg orally twice a day 5 days a week for three weeks had minimal effect on tumor growth. However, KPT-330 given orally at 10 mg/kg twice a week for three weeks could suppress the growth of 8505C xenograft significantly (*p* < 0.05). KPT-9274, given at 100 mg/kg twice a day 5 days a week for 3 weeks, showed slight reduction in tumors. Remarkably, when combined with lenvatinib, both KPT-330 and KPT-9274 could reduce tumor growth to a greater extent compared to single-agent treatments (Figure 6A,B). There was no notable body weight loss in the mice during the course of treatment (Figure 6C). Gross tumor weight showed statistically significant reduction in KPT-330 + lenvatinib group compared to single-agent lenvatinib (Figure 6D). Furthermore, the combination of lenvatinib with either KPT-330 or KPT-9274 resulted in superior reduction of ki67 proliferation marker in tumor tissue (Figure 6E,F). We also evaluated the changes in the gene expression of *Vimentin* post treatment. As can be seen from the results of Figure 6G, compared to single-agent treatment, the combination of lenvatinib with either KPT-330 or KPT-9274 caused greater inhibition of EMT marker *Vimentin*. These results support the use of KPT-330 or KPT-9274 either as single agents or in combination with lenvatinib for therapy resistant thyroid cancer.

## 3. Discussion

In this paper we show that long term culturing of thyroid cancer cell line 8505C in lenvatinib causes marked genomic and phenotypic changes that indicate a shift towards mesenchymal cell morphology. Our RNA-Seq analysis in the resistant cells identifies actionable avenues that can be targeted using novel combinations which show activity in vitro and in vivo.

While the treatment outcomes for most thyroid cancer patients are excellent, about 10% of patients do not respond to surgery, radioactive iodine, and thyroid hormone suppression. In such refractory patients, tyrosine kinase inhibitors (TKI) are emerging as significant game changers and show efficacy and potency [11]. Lenvatinib, a TKI drug, shows activity against refractory thyroid cancer. A phase II study of lenvatinib conducted in Japan has documented a median OS in patients with radioactive refractory DTC at 31.8 months (95% CI: 31.8-NR), 12.1 months (95% CI: 3.8-NR) in patients with MTC and 10.6 months (95% CI: 3.8-19.8) in patients with ATC [12]. Despite this success, a proportion of patients become refractory to lenvatinib and have no alternative therapies. Additionally, although lenvatinib can delay progression of thyroid cancer, patients receiving the drug presented AEs that include hypertension (in 68% of the patients), diarrhea (59%), fatigue or asthenia (59%), decreased appetite (50%), decreased weight (46%) and nausea (41%) [10]. Therefore, it is imperative to understand the underlying mechanisms of resistance to lenvatinib and design less toxic combination strategies to treat refractory thyroid cancer. 

The nuclear exporter protein XPO1 guides the movement of different proteins from the nucleus to the cytoplasm in an energy dependent process involving binding to guanine exchange factors [13]. The export of cargo occurs through a precise interaction with the receptor (XPO1) that recognizes the conserved nuclear export signal (NES) in different proteins. Unusual export of nuclear tumor suppressors and genome surveillance proteins, as observed in most cancers, disturbs this balance [13]. As a consequence, the mislocalized tumor suppressor proteins (TSPs) get functionally inactivated. Studies have shown that targeted inhibition of XPO1 by selective inhibitors of nuclear export (SINE) compounds can restore the nuclear TSP function leading to inhibition of tumor growth [14]. The lead SINE compound KPT-330, also known as selinexor, has shown broad activity in several Phase I/II/III clinical trials in patients with solid tumors and hematological malignancies. Selinexor has recently received FDA approval for penta-refractory multiple myeloma [15]. In the context of thyroid cancer, studies have shown that XPO1 and related exportins influence thyroid hormone receptor nuclear export and function [16,17]. Interestingly, in a distinct study, selinexor was shown to overcome TKI resistance in a leukemia model [18]. Moreover, a recent study from our lab has demonstrated that XPO1 may act as a promising molecular target in gastric cancer and blocking it using SINE compounds can have therapeutic significance [19]. In alignment to these studies, our RNA seq analysis showed activation of cargo receptor activity and nuclear pore signaling molecules in the resistant thyroid cell line. This led us to explore the combination of nuclear export inhibitors KPT-330 (selinexor) and next generation compound KPT-8602 (eltanexor) with lenvatinib. Indeed, our results show that selinexor can synergistically enhance the anti-tumor activity of lenvatinib in vitro and in vivo.

P21 activated kinase (PAK) family proteins are downstream effectors of Rho GTPases that promote cell proliferation, adhesion, motility, plasticity, resistance, and stemness [20]. PAK4 has been shown to play significant role promoting resistance and stemness in solid tumors [21]. In a recent study PAK4 was shown to be involved in TSH induced papillary thyroid cancer cell proliferation [22]. Indeed, our analysis of the biological pathways showed statistically significant changes in adhesion pathways, cell cycle pathways, regulatory signaling, locomotion, motility, metabolic processes among others that are influenced by PAK4 signaling. More significantly, independent studies have shown that MAPK and AKT activated thyroid cancers are responsive to PAK4 inhibition [23]. This prompted us to test the combination of PAK4 inhibitor KPT-9274 with lenvatinib. As anticipated, we observed synergy between KPT-9274 and lenvatinib in vitro. The anti-tumor response was markedly enhanced with this combination in thyroid cancer subcutaneous xenograft. More significantly, both KPT-330 and KPT-9274 were used at sub-optimal doses and did not cause any significant body weight loss or other signs of outward toxicity indicating that the combination was well tolerated.

Therefore, it may be envisaged that targeting XPO1 by SINE or PAK4 by KPT-9274 may serve to reduce the dose and frequency of lenvatinib regimen required to achieve therapeutic effects in anaplastic thyroid cancer. It is plausible that such a reduction in lenvatinib dose regimen would decrease the chances or delay the onset of lenvatinib therapy resistance in thyroid cancer patients. However, it warrants further investigations to establish that KPT-330 or KPT-9274 may complement lenvatinib in a clinical setting. Nonetheless, we have demonstrated the therapeutic relevance of targeting nuclear export protein XPO1 and Rho GTPase effector PAK4 which has possible implications in overcoming lenvatinib therapy resistance in anaplastic thyroid cancer.

## 4. Materials and Methods

### 4.1. Cell Line, Culture Conditions and Reagents

The human ATC cell line 8505C was obtained from Sigma (St. Louis, MO, USA). The cell line was authenticated using short tandem repeat at the Karmanos Cancer Institute. Cells were cultured in a mixture of Dulbecco’s Modified Eagle Medium (DMEM) with 10% FBS and penicillin and streptomycin. For the development of lenvatinib resistant cell line, 8505C cells were cultured in a medium containing 25 µM lenvatinib for a period of 72 days. Karmanos Cancer Institute clinic provided lenvatinib (Eisai Inc., Woodcliff Lake, NJ, USA). KPT-330 (selinexor/XPOVIO), KPT-8602 (eltanexor) and KPT-9274 were obtained from Karyopharm Therapeutics Inc. (Newton, MA, USA). For in vitro work, KPT-330, KPT-8602 or KPT-9274 were dissolved in DMSO to make 50 mM stocks and used in serial dilution for different assays. Similarly, a 1 mM stock solution of lenvatinib was also prepared in DMSO. 3-(4,5-dimethylthiazol-2-yl)-2,5-diphenyltetrazolium bromide (MTT) was obtained from Sigma (St. Louis, MO, USA). All the primary antibodies used for Western blot analysis were obtained from Cell Signaling Technology (Danvers, MA, USA).

### 4.2. Cell Growth Inhibition by MTT Assay

Cells were seeded at a density of 5 × 10^3^ cells per well in 96-well micro-titer culture plates. After overnight incubation, the medium was removed and replaced with fresh medium containing the respective drugs at various concentrations. After 72 hours of incubation, MTT assay was performed by adding 20 µL of MTT solution (5 mg/mL in PBS) to each well and incubated further for 2 hours. Upon termination, the supernatant was aspirated and the MTT formazan formed by metabolically viable cells was dissolved in 100 µL of DMSO. The plates were gently rocked for 30 minutes on a gyratory shaker, and absorbance was measured at 595 nm using a plate reader (TECAN, Durham, NC, USA). 

### 4.3. Quantification of Apoptosis by Annexin V FITC Assay

Cell apoptosis was detected using Annexin V FITC (Biovision, Danvers, MA, USA) according to the manufacturer’s protocol. Cells were seeded at a density of 50,000 cells per well in six-well plates in 5 mL of corresponding media. 24 h after seeding, the cells were exposed to 25 µM lenvatinib for 72 h. At the end of the treatment period, cells were trypsinized and equal numbers were stained with Annexin V and Propidium Iodide. The stained cells were analyzed using a Becton Dickinson (Franklin Lakes, NJ, USA) flow cytometer at the Karmanos Cancer Institute Flow Cytometry core.

### 4.4. RNA Isolation and mRNA Real-Time RT-qPCR 

Total RNA from cell lines or residual tumor tissues was extracted and purified by using the RNeasy Mini Kit and RNase-free DNase Set (QIAGEN, Valencia, CA, USA) following the protocol provided by the manufacturer. The mRNA expression of various markers was analyzed by real-time RT-qPCR using High Capacity cDNA Reverse Transcription Kit and SYBR Green Master Mixture from Applied Biosystems (Waltham, MA, USA). Sequences of primers used are listed in Table 2. The qPCR was initiated by 10 min at 95 °C before 40 thermal cycles, each of 15 s at 95 °C and 1 min at 60 °C in a StepOnePlus real-time PCR system (Applied Biosystems, Waltham, MA, USA). Data were analyzed according to the comparative Ct method and were normalized to actin and/or GAPDH rRNA expression in each sample.

### 4.5. Preparation of Total Protein Lysates and Western Blot Analysis

For total protein extraction, cells were lysed in RIPA buffer and protein concentration was measured using BCA protein assay (PIERCE, Rockford, IL, USA). Western Blot analysis was conducted to measure the alterations in the protein expression of genes. Briefly, the total proteins were subjected to 10% or 14% SDS-PAGE, and electrophoretically transferred to nitrocellulose membrane. The membranes were incubated with specific primary antibodies, and subsequently incubated with secondary antibody conjugated with peroxidase (Bio-Rad, Hercules, CA, USA). The signal was detected using the chemiluminescent detection system (PIERCE, Rockford, IL, USA). Densitometric analysis of the data was performed using the ImageJ software (version java 8; NIH, Bethesda, MD, USA).

### 4.6. RNA Sequencing

Total RNA from each sample was extracted and purified by using the miRNeasy Mini Kit and RNase-free DNase Set (QIAGEN, Valencia, CA, USA) following the protocol provided by the manufacturer. The RNA samples were sent to LC Sciences (Houston, TX, USA) and subjected to poly(A) RNA sequencing. In their laboratory, the total RNA quantity and purity were analyzed by Bioanalyzer 2100 and RNA 6000 Nano LabChip Kit (Agilent, Santa Clara, CA, USA). Ten micrograms of total RNA were subjected to isolation of Poly(A) mRNA with poly-T oligo attached magnetic beads (Invitrogen, Carlsbad, CA, USA). Following purification, the poly(A)- or poly(A)+ RNA fractions are fragmented into small pieces. The cleaved RNA fragments were reverse-transcribed to create the final cDNA library. Then, the paired-end sequencing on an Illumina Hiseq 4000 was performed following the manufacturer recommended protocol. The mapped reads of each sample were assembled using StringTie. All transcriptomes from samples were merged to reconstruct a comprehensive transcriptome. After the final transcriptome was generated, the expression levels of all transcripts were calculated. The differentially expressed mRNAs were selected with log2 (fold change) > 1 or < -1 and with statistical significance (*p*-value < 0.05) by R package Ballgown. The bioinformatics tool used to perform the gene annotation analysis and to assess molecular functions, biological processes and pathways was PANTHER v.14.0 (http://www.pantherdb.org). The Protein ANalysis THrough Evolutionary Relationships (PANTHER) classification system is a comprehensive system that combines genomes, gene function classifications, pathways and statistical analysis tools to analyze large-scale genome-wide experimental data [24].

### 4.7. Animal Xenograft Studies

All animal studies were performed under a Wayne State University IACUC (Institutional Animal Care and Use Committee) approved protocol (#18-12-0887; 17 April 2019). ICR SCID mice were inoculated with 8505C cells in the flank. Once tumors were established, they were trocared in 30 different mice. Palpable tumors were observed in 26 mice. These tumor bearing animals were then randomly divided into six different groups, with the two combination treatment groups receiving five mice each while the rest of the groups received four mice each. Group 1: control; Group 2: lenvatinib 50 mg/kg twice a day 5 days a week for 3 weeks; Group 3: KPT-330 (selinexor) 10 mg/kg orally twice a week for 3 weeks; Group 4: selinexor 10mg/kg orally twice a week for 3 weeks + lenvatinib 50 mg/kg twice a day 5 days a week for 3 weeks; Group 5: KPT-9274 at 100 mg/kg twice a day orally 5 days a week for 3 weeks and Group 6: KPT-9274 at 100 mg/kg twice a day orally 5 days a week for 3 weeks + lenvatinib 50 mg/kg twice a day 5 days a week for 3 weeks. Mice tumors were measured every three days. Tumor volume was calculated using the formula (length × width^2^)/2. At the end of the treatment the mice were humanely sacrificed and tumors were harvested.

## 5. Conclusions

In conclusion, our studies bring forward two novel targets XPO1 and PAK4 as actionable therapeutic avenues to overcome resistance to lenvatinib in refractory anaplastic thyroid cancer.

## Figures and Tables

**Table 1:** The gene alterations indicative of resistance.

Gene_id	Gene_Name	8505 C	8505 Res	Regulation
ENSG00000128052	*KDR (VEGFR)*	0.05	0.12	up
ENSG00000148516	*ZEB1*	2.65	4.81	up
ENSG00000124216	*SNAI1*	2.36	2.71	up
ENSG00000019549	*SNAI2 (Slug)*	1.81	4.80	up
ENSG00000140379	*BCL2A1*	1.42	11.18	up
ENSG00000042832	*TG (Thyroglobulin)*	0.01	0.01	-
ENSG00000079385	*CEACAM1*	0.57	0.61	up
ENSG00000105352	*CEACAM4*	0	0.07	up
ENSG00000105388	*CEACAM5*	0.31	0.44	up
ENSG00000086548	*CEACAM6*	0.66	0.76	up
ENSG00000176046	*NUPR1*	1.29	13.48	up

**Table 2:** Sequences of primers used.

Primers	Sequences	
MCL1	Forward	TTCCAGTAAGGAGTCGGGGT
	Reverse	CCTCCTTCTCCGTAGCCAAAA
Bcl-2	Forward	TGAACTGGGGGAGGATTGTG
	Reverse	CGTACAGTTCCACAAAGGCA
Bax	Forward	AGGTCTTTTTCCGAGTGGCA
	Reverse	CCCGGAGGAAGTCCAATGTC
PI3K	Forward	GAGCCCCGAGCGTTTCTG
	Reverse	TCGTGGAGGCATTGTTCTGA
Akt	Forward	TTGTGAAGGAGGGTTGGCTG
	Reverse	CTCACGTTGGTCCACATCCT
mTOR	Forward	TTCCGACCTTCTGCCTTCAC
	Reverse	CCACAGAAAGTAGCCCCAGG
EGF	Forward	CTGAATGTCCCCTGTCCCAC
	Reverse	CTCGGTACTGACATCGCTCC
Pak1	Forward	CCCCTTGGACTCTCATTCCC
	Reverse	GAGGCAGGAGGTGGTAACTG
Pak4	Forward	GTGCAAGAGAGCTGAGGGAG
	Reverse	ATGCTGGTGGGACAGAAGTG
XPO1	Forward	GGAAAACTGTGAAACCCACCTT
	Reverse	GCTGCATGGTCTGCTAACAT
Vimentin	Forward	GGACCAGCTAACCAACGACA
	Reverse	AAGGTCAAGACGTGCCAGAG
Snail	Forward	GGCCTGGGAGGAAGATGTTTAC
	Reverse	CCCCTCCTCCCTTACCAAAGA
Nanog	Forward	GAAATACCTCAGCCTCCAGCA
	Reverse	TTCTGCGTCACACCATTGCTA
ALDH	Forward	CCAGGGCCGTACAATACCAA
	Reverse	GTGCAGGCCCTATCTTCCAA
actin	Forward	GCACAGAGCCTCGCCTT
	Reverse	TCATCATCCATGGTGAGCTG
GAPDH	Forward	GGAGAGTGTTTCCTCGTCCC
	Reverse	ATGAAGGGGTCGTTGATGGC

## Supplementary Materials

Supplementary materials can be found at https://www.mdpi.com/1422-0067/21/1/237/s1.
