# Supplementary material for: Targeting XPO1 and PAK4 in 8505C Anaplastic Thyroid Cancer Cells: Putative Implications for Overcoming Lenvatinib Therapy Resistance
Source: Int J Mol Sci. 2019 Dec 29;21(1):237. doi: 10.3390/ijms21010237 (PMC6982268; doi:10.3390/ijms21010237)
Supplement: Supplementary file 1 [file ijms-21-00237-s001.pdf]

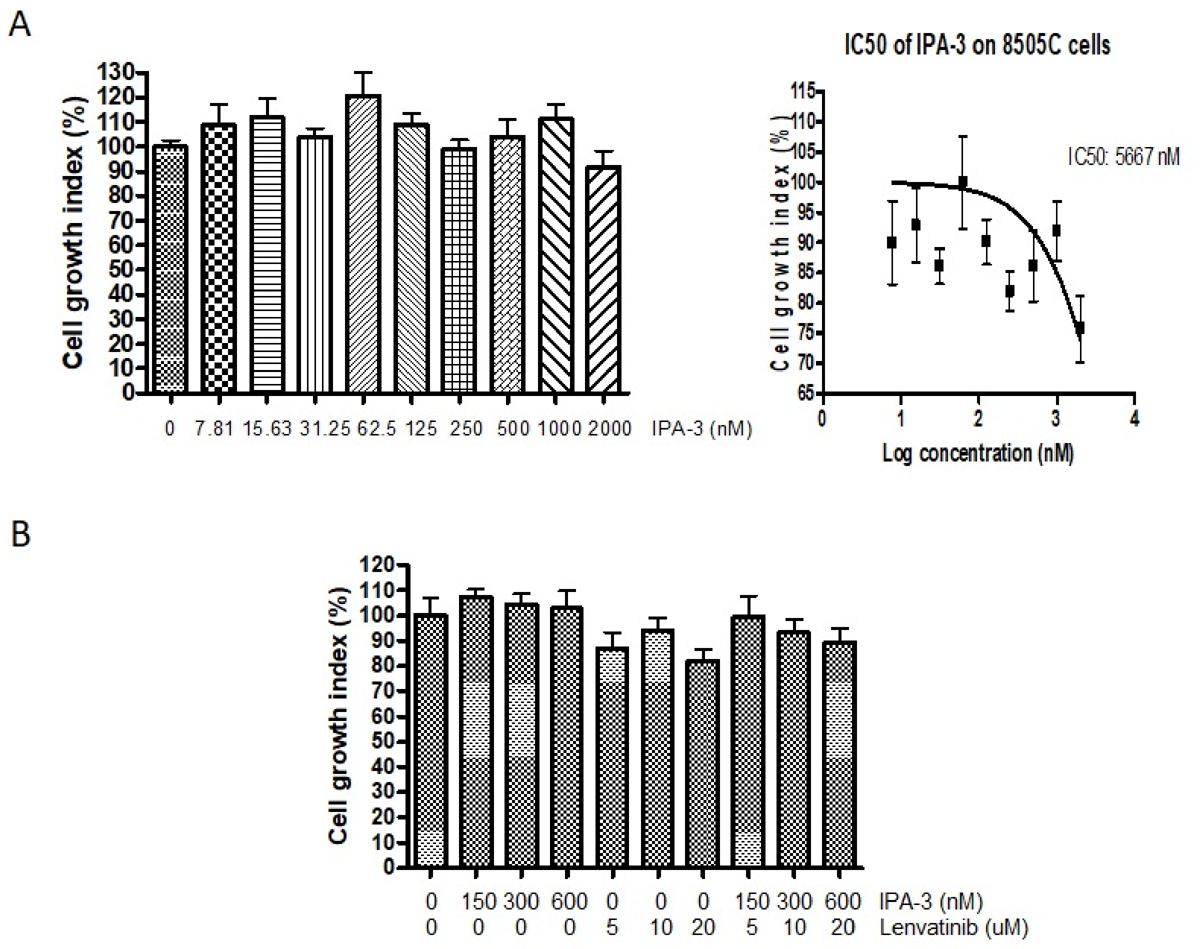

Supplementary Figure S1. Inhibitor of PAK1 does not suppress anaplastic thyroid cancer cell proliferation neither alone nor in combination with lenvatinib. [A] 8505C cells were seeded in 96 well plates at a density of 5000 cells per well. The next day cells were exposed to increasing concentrations of IPA-3 for 72 hrs. At the end of treatment, MTT assay was performed according to standard procedure described in methods section. Each point represents six replicates. [B] 8505C thyroid cancer cells were treated with indicated concentrations of either lenvatinib or IPA-3 or combinations of lenvatinib with IPA-3 for 3 days. Cell proliferation assay using MTT was conducted.
